# Sterilization, Infecundity, and Reproductive Autonomy in Rural, Suburban, and Urban America: Results From a National Survey

**DOI:** 10.1111/psrh.70006

**Published:** 2025-03-16

**Authors:** Shelley Clark, Zoe Levy

**Affiliations:** ^1^ McGill University Montreal Canada

## Abstract

**Objective:**

Rural women are significantly more likely than urban women to be sterilized. This study aims to understand why rural women depend so heavily on this method of fertility control, whether they are more likely than suburban and urban women to desire sterilization reversal, and the impact of female sterilization on rural women's ability to achieve their fertility goals.

**Methods:**

Data from 10,081 sexually active women aged 15 to 49, who participated in the National Survey of Family Growth (2015–2019), were analyzed using binary and multinomial logistic regression analyses. Unadjusted and adjusted predicted probabilities were calculated to estimate the prevalence of (1) female sterilization, (2) desire for sterilization reversal or wanting a(nother) child if sterilized, and (3) unwanted infecundity among rural, suburban, and urban women.

**Results:**

Rural women (24.2%) are substantially more likely than suburban (15.3%) or urban (13.9%) women to receive tubal ligation. These disparities are not explained by women's demographic, reproductive, religious, and socioeconomic characteristics. Rural women who are sterilized are not more likely than suburban or urban women to desire sterilization reversal or to want to have (more) children. However, because more rural women rely on tubal ligation, a significantly higher fraction of rural women (34.8%) than urban women (23.8%) who want to have a(nother) child are infecund. Roughly, 40% of infecund rural women who wish to conceive had tubal ligation.

**Conclusions:**

Limited contraceptive choice undermines rural women's ability to conceive wanted births. These results highlight another important reason for expanded reproductive health care in rural America.

## 1 Introduction

Rural America has limited access to contraception, abortion care, and reproductive health services, as compared to urban and suburban areas in the United States [1, 2, 3]. These disparities can undermine rural women's reproductive autonomy in a myriad of ways. Research thus far has primarily focused on how unequal access results in higher levels of unintended pregnancies and births among rural women, and specifically among teenagers and young adults [4, 5, 6, 7]. Less considered is whether these disparities undermine rural women's reproductive autonomy by causing an over‐reliance on contraceptive methods that limit their ability to have wanted pregnancies in the future.

Women may be more likely to seek permanent methods of contraception when contraceptive choices are constrained and access to abortion is restricted. This may help explain why female sterilization, specifically tubal ligation, is nearly twice as common among rural women than urban women [8, 9, 10, 11, 12].^1^ Although tubal ligation offers women many advantages, including that the procedure is safe [13], highly effective, relatively affordable, and does not require repeated visits to a health care provider or pharmacy, it has one notable drawback. It is difficult, and often expensive, to reverse [14, 15]. Given that fertility goals often change [16, 17], heavy reliance on female sterilization may result in a substantial proportion of rural women who find that they are unable to have a child when they want one.

This study examines the factors associated with the higher rates of female sterilization in rural areas compared to either suburban or urban areas. It further explores whether sterilized rural women are more likely than suburban or urban women to want to have their procedure reversed or to have a(nother) child. Lastly, it assesses the implications that heavy reliance on sterilization may have on rural women's reproductive autonomy, specifically their ability to have (additional) wanted children.

## 2 Female Sterilization in Rural America

Nationally representative data from 2006 to 2010 show that nearly twice as many rural women (22.8%) compared to suburban and urban women (12.7%) are sterilized [12]. Although reasons for these differences are not well understood, personal characteristics, including age, marital status, parity, and previous unintended pregnancy as well as socioeconomic status (SES), religious beliefs, and race and ethnicity, may influence the use of female sterilization [12, 18, 19, 20, 21, 22]. Since these characteristics differ among rural and urban women, they could explain higher rural uptake of sterilization. For example, rural women tend to be slightly older and to both marry [23] and have children at younger ages [23, 24]. Rural women are also more likely than urban women to have lower levels of education [25] and lower incomes [26], to hold more conservative or fundamentalist religious views, and to have previously experienced unintended pregnancy [6]. Given that these characteristics have been positively associated with seeking tubal ligation, they may partially explain the higher levels of sterilization among rural women.

Rates of tubal ligation also differ by race and ethnicity, reflecting histories of coerced sterilization targeting Black, Hispanic, Indigenous, and poor women [27, 28]. As late as 2002, Black and Hispanic women in the United States reported being significantly more likely than non‐Hispanic White women to use tubal ligation [14, 29]. More recent studies, however, do not show significant racial disparities in female sterilization after controlling for background characteristics [19, 30, 31]. Although urban areas in the United States are more racially and ethnically diverse than rural populations, declining racial inequalities in sterilization may result in starker rural–urban sterilization disparities.

In addition to personal characteristics, barriers to access or limited availability of other contraceptive options may contribute to a disproportionate use of sterilization among rural women. For example, rural women are less likely than urban women to use long‐acting reversible contraceptives (LARCs), such as IUDs and hormonal implants, possibly because rural healthcare providers are less likely to have LARC devices available [32] and are less familiar with insertion and removal techniques [3, 33]. Given that prior use of LARC is associated with a lower likelihood of sterilization, the availability of LARCs may present an important alternative to sterilization [34]. Rural women are also less likely to use or to receive counseling about emergency contraception [35]. Lastly, rural women may face greater barriers to accessing abortion services. Emerging studies provide compelling evidence that when access to abortion is restricted, female sterilization increases [36, 37].

Where women seek reproductive health care and access to health insurance may also impact their likelihood of choosing sterilization. Rural women are more likely to seek care from a family doctor or nurse practitioner than from an obstetrician‐gynecologist (OBGYN) or family planning specialist [38]. Women with either public or no health insurance are also more likely than women with private insurance to be sterilized [14, 39]. Given that rural women are more likely than urban women to be uninsured [38], health insurance status may also partially explain differences in sterilization between rural and urban women.

## 3 Desire for Sterilization Reversal or Wanting A(nother) Child Among Sterilized Women

Several previous studies have examined whether women who have had tubal ligation subsequently “change their minds” or “regret” their contraceptive decisions [11, 12, 40, 41, 42, 43, 44].

These studies show that after sterilization, a sizable fraction of sterilized women subsequently express a desire to have a(nother) child or to have the sterilization procedure reversed. For example, the percentage of women who want a tubal ligation procedure reversed typically ranges between 20% and 30% [40, 41, 42]. Both women's current age and their age at the time of the procedure may be important predictors of wanting the procedure reversed and desire for (more) children [41, 42, 43, 45]. Women's union status [42], number of children [42], race/ethnicity [40, 43, 44, 46], and education [45] may also be associated with a desire for sterilization reversal, although findings are often mixed. Recent research further suggests that the desire for sterilization reversal is associated with unknown and generally unforeseeable life events such as current unions ending and new ones forming [42].

It is unclear whether place of residence also affects the likelihood of wanting sterilization reversal or wanting a(nother) child, if sterilized. We were able to locate only two prior studies that tested for differences in wanting to have the procedure reversed among women living in rural or urban areas. Neither study found a significant difference, although one study noted this may have been due to the small sample size of its rural population [11, 12]. Further, both studies draw on data collected more than a decade ago, and one study is limited to a single state. Nonetheless, even if rural women are no more likely than urban women to desire sterilization reversal, given that a larger fraction of rural women have been sterilized, more rural than urban women may find that they are unable to conceive if they want to have a(nother) child.

## 4 Unwanted Infecundity

In this paper, unwanted infecundity refers to women who want to have a(nother) child but who are biologically or medically unable to have one. There are two main ways to measure unwanted infecundity that yield relevant insights and have important implications for health practitioners, policymakers, and researchers. One measure of infecundity captures the proportion of all women who both want to have a(nother) child and are infecund. This proportion can assist health providers in estimating the number of women who may be interested in reproductive assistance technologies. However, this measure of unwanted infecundity reflects both levels of infecundity and fertility desires, which may differ between rural and urban women. More rural women than urban women may want to have a(nother) child if rural women want, on average, more children. Conversely, a higher proportion of urban women may want a(nother) child if urban women are more likely to delay childbearing. Another measure of unwanted infecundity, which removes the effect of differences in fertility desires, assesses infecundity among women who want to have a(nother) child. This measure provides important insights into women's reproductive autonomy by assessing the alignment between women's fertility goals and their ability to achieve them.

The sources of infecundity may also differ across rural, suburban, and urban women. A variety of health conditions, along with advancing age, can compromise women's ability to get pregnant, rendering them subfecund or infertile. Infecundity refers to the inability of women to conceive or carry a pregnancy to term. Subfecundity usually refers to conditions that make it physically difficult for a women to conceive or deliver a pregnancy, while infertility typically refers to otherwise fecund women who report no pregnancy over an extended period of time despite constant exposure to risk [47]. Women may also be infecund because they have undergone surgically sterilizing operations such as tubal ligation, hysterectomies or bilateral ovary removal for both contraceptive and non‐contraceptive reasons. In some instances, women may be able to conceive or carry a pregnancy to term, but their male partners may be sterile if they have had a vasectomy or other health conditions.

The extent to which tubal ligation contributes to unwanted infecundity is largely unknown. It will depend on both the proportion of women who have had tubal ligation and the proportion of sterilized women who want to have a(nother) child but have not had the procedure reversed. Although up to a quarter of sterilized women indicate that they want to have their tubal ligation reversed, relatively few do so [41, 42]. Increasingly, women who have had tubal ligation opt for IVF rather than reversal, although both methods have success rates of less than 60% and carry risks and sizeable costs [48]. This suggests that many women who have had tubal ligation may feel it is too late or too expensive to change their minds. Rural women may not only be more likely to have tubal ligation, but they may also face greater geographic and financial barriers to accessing procedures to reverse their sterilization or IVF services. Consequently, female sterilization may play a key role in rural unwanted infecundity and prevent a disproportionate number of rural women who want to have (more) children from having them.

This study has three main objectives. Drawing on data from the National Survey of Family Growth (2015–2019) [49], we first examine why female sterilization is higher in rural areas than in suburban or urban areas. Second, we test whether sterilized rural women are more likely than suburban or urban women to want to have their procedures reversed or to want a(nother) child. Third, we determine whether heavy reliance on female sterilization has the unintended consequence that infecundity is higher among rural women who want to have (more) children.

## 5 Data and Methods

### 5.1 Data and Sample

For our analyses, we pool publicly available data from two waves of the National Survey of Family Growth (NSFG) 2015–2017 and 2017–2019.^2^ This yields a combined nationally representative sample of 10,122 sexually active women between the ages of 15 and 49 (inclusive).^3^ The female response rate was 66.7% in the 2015–2017 wave and 62.5% in 2017–2019 wave [50]. Women responded to in‐depth questions about their pregnancy histories, use of contraception, prior sterilization procedures for themselves and their partners, future fertility desires as well as their background demographic, reproductive, religious, and socioeconomic characteristics. For our analyses of female sterilization, we removed 41 women (0.4%) who had missing information on any of these characteristics, yielding an analytic sample of 10,081 women. Of these women, 1525 had a tubal ligation for contraceptive purposes and have not had it reversed.^4^ We draw on this sample of 1525 women who had a tubal ligation to examine both the desire to have a(nother) child and the desire to have the procedure reversed. Women who had at least one other sterilization procedure (i.e., hysterectomy, bilateral ovary removal) (*n* = 265) were not asked the question about whether they wished to have their tubal ligation procedure reversed and, hence, are excluded from these analyses, yielding an analytic sample of 1260. To examine unwanted infecundity, we first estimate the percentage of all women who both want a(nother) child and are infecund (*n* = 10,081). We then examine infecundity among a subsample of women who want to have a(nother) child (*n* = 5217).

### 5.2 Measures

Our analyses examine three primary outcomes. First, we generate a dichotomous variable to indicate whether the woman is currently surgically sterile due to tubal ligation. Second, we examine whether women who had unreversed tubal ligation expressed the desire to have a(nother) child or to have their sterilization procedure reversed. To measure future fertility desires, we used responses to the question “Looking to the future, (if it were possible) do you, yourself, want to have (a/nother) baby at some time in the future?” In addition, we examined responses to the question, “As things look to you now, if your tubal sterilization could be reversed safely, would you want to have it reversed?” For both questions, we coded women who responded “definitely yes” or “probably yes” as desiring a(nother) child/sterilization reversal. Our third outcome measures infecundity. Women are considered to be infecund if they report any of the following conditions: had a tubal ligation (that was not reversed), had different sterilizing surgery (such as a hysterectomy or bilateral ovary removal) or are infertile (unable to conceive for more than 36 months) or subfecund (have difficulty conceiving or gestating). Women are also considered infecund if their cohabiting or marital male partner has had a vasectomy.

Our primary independent variable is whether the woman lives in a rural, suburban, or urban area. The NSFG uses the Office of Management and Budget classification scheme to indicate whether the respondent lives in (1) the principal city of a metropolitan statistical area (MSA), (2) other MSA, or (3) a non‐MSA county at the time of the interview. Counties are classified according to their 2010 Census population counts. Following conventional practice [6], we relabel these categories as urban, suburban, and rural, respectively.

Our analyses adjust for several personal characteristics. Given the low percentage of women younger than age 30 who have had tubal ligation (< 3%), we grouped respondents into ages 15–29, 30–34, 35–39, 40–44, and 45–49. Race and ethnicity are measured as “1” non‐Hispanic White, “2” non‐Hispanic Black, “3” Hispanic or Latinx, and “4” non‐Hispanic other. An indicator variable for immigrant status equals “1” if the respondent was born outside the United States and “0” otherwise. Women's union status reflects whether the woman is currently married, cohabiting with a partner, formerly married (divorced, separated, or widowed), or never married. We also include two measures of women's previous reproductive histories: her current number of children (< 2, 2, 3, and ≥ 4 children)^5^ and whether she has ever experienced an unwanted pregnancy. An additional measure of women's reproductive histories is added to our models of sterilized women who want a(nother) child or desire to have their sterilization reversed. For these analyses, we also adjust for the age at which women had their tubal ligation procedure. In all our models, women's SES is measured by her highest level of education (less than high school, high school degree, some college, and college degree or higher), and her household income (< $25,000, $25,000– < $50,000, $50,000– < $75,000, and ≥ $75,000). Women's current religious affiliation is categorized as “1” no religion, “2” Christian, non‐fundamentalist, “3” Christian, fundamentalist, and “4” other religions. Christian fundamentalists refer to Christians who identify as being evangelical or born again. In addition, we capture differences in health care access through women's type of health insurance (not covered, public (including both Medicare and Medicaid), and private) and women's usual place of health care (no usual place, HMO or private doctor, health clinic, or emergency care).

### 5.3 Analysis

We first estimate descriptive statistics (weighted proportions and means) of rural, suburban, and urban women's characteristics. Adjusted Wald tests are used to evaluate differences by residence. Binary logistic regressions are then used to estimate the probability of (1) tubal ligation among all sexually active women and (2) desire for having a(nother) child and desire for sterilization reversal among women who had an unreversed tubal ligation. We examine our third outcome, unwanted infecundity, with two measures. First, we compare the percentage of all rural, suburban, and urban women who both want a(nother) child and are infecund from any cause. We then use binary logistic regression to assess differences in infecundity from any cause among women who want a(nother) child.

Lastly, to further investigate the extent to which differences in tubal ligation are driving rural‐suburban‐urban differences in infecundity, multinomial logistic regressions are used to compare infecundity due to tubal ligation and due to all other causes of infecundity (except tubal ligation) among women who want a(nother) child. Our first model provides the unadjusted predicted probabilities and average marginal effects of suburban and urban women compared to rural women. Model 2 subsequently controls for women's characteristics. Full results from the adjusted models are shown in Tables S1–S3. All analyses are performed in Stata 18 and implement svy (survey) commands to account for population weights and survey design. As this study draws on publicly available data, institutional review board approval was not required.

## 6 Results

### 6.1 Descriptive Characteristics

Table 1 shows the demographic, reproductive, religious, and socioeconomic characteristics of rural, urban, and suburban women. Age differences by residence are small, ranging from an average of 33.1 years among urban women to 34.2 years among suburban women. Marriage is most common among suburban women (51.5%), while rural women are most likely to be in a cohabiting partnership (19.8%) and urban women are most likely to have never been married (35.7%). Urban women are more likely than either rural or suburban women to have less than two children, but there are no statistically significant differences in the average number of children by residence. There are also no differences in the likelihood of reporting a previous unwanted pregnancy. However, among sterilized women, suburban women are sterilized at slightly older ages than either rural or urban women. Urban women are the most racially and ethnically diverse, with less than half (48.4%) identifying as White, compared with 62.9% of suburban women and 76.4% of rural women.

**TABLE 1:** Descriptive characteristics of sexually active women aged 15–49 by residence, National Survey of Family Growth 2015–2019.

Variable	Residence			Significance		
	Rural	Suburban	Urban	Rural versus suburban	Rural versus urban	Urban versus suburban
*n*	1619	4564	3898			
Age at interview (mean)	33.9	34.2	33.1			**
Race/Ethnicity (%)						
White	76.4	62.9	48.4	**	***	***
African America	11.8	12.2	21.2		*	***
Hispanic	9.0	18.8	24.0	**	***	*
Other	2.8	6.2	6.4	***	***	
Immigrant (%)	8.2	17.6	18.6	***	***	
Union Status						
Currently married	43.9	51.5	37.0	**	*	***
Cohabiting	19.8	12.7	16.3	***		**
Formerly married	12.1	10.4	10.9			
Never married	24.2	25.3	35.7		***	***
Parity (mean)						
0–1 Children	51.1	52.3	57.6		*	*
2 Children	27.7	25.6	22.0		*	*
3 Children	15.4	15.1	12.8			
≥ 4 Children	5.8	7.0	7.6			
Prior unwanted pregnancy (%)	21.1	18.5	21.4			
Age at sterilization (mean)^a^	28.2	30.4	29.0	**		*
Education (%)						
Less than high school	19.4	12.9	15.8	**		
High school	25.6	19.5	19.6	*	*	
Some college	31.9	29.9	28.8			
College or higher	23.1	37.7	35.8	***	***	
Household Income (%)						
< $25,000	32.6	20.0	29.0	***		***
$25,000– < $50,000	25.7	22.5	27.2			**
$50,000– < $75,000	19.5	19.0	17.8			
≥ $75,000	22.2	38.5	26.1	***		***
Religious affiliation (%)						
No religion	23.6	23.5	28.2			*
Christian, non‐fundamentalist	33.5	44.8	40.9	**	*	
Christian, fundamentalist	40.9	27.1	24.8	**	***	
Other religion	2.0	4.7	6.1	***	***	
Health Insurance (%)						
Private	58.6	68.7	58.9	***		***
Public	26.1	20.6	26.8			**
Not covered	15.4	10.7	14.2			*
Usual place for health care (%)						
None	12.5	12.6	15.2			
HMO/Private doctor	63.2	67.6	58.8			***
Clinic	16.4	13.3	18.6			***
Emergency care	7.9	6.5	7.5			

*Note:* All percentages and means are weighted. Total unweighted analytic sample of 10,081 respondents. Significance: *p ≤ 0.05, **p ≤ 0.01, ***p ≤ 0.001.

^a^ Limited to 1525 women who had an unreversed tubal ligation procedure.

In general, suburban women tend to have the highest SES, while rural women have the lowest. For example, 37.7% of suburban women have earned a college degree compared to only 23.1% of rural women. The SES of urban women tends to fall in between. More than two‐thirds of women in the sample identify as Christian, but rural women are significantly more likely than either suburban or urban women to report holding fundamentalist beliefs such as being born again. Rural and urban women have nearly identical sources of health insurance, while suburban women are significantly more likely than either to have private health insurance. Suburban women are also more likely than urban women to seek health care via their HMO or a private doctor rather than at a clinic.

### 6.2 Female Sterilization

Model 1 of Table 2 presents the predicted probabilities and average marginal effects for whether women are sterilized by residence. We find that rural women are 1.7 times more likely to be sterilized than urban women and 1.6 times more likely than suburban women (24.2% rural vs. 13.9% urban and 15.3% suburban). Differences in rural, suburban, and urban women's characteristics explain some, but not all, of these differences in sterilization (Model 2). In our unadjusted model (Model 1), rural women are nine percentage points more likely than suburban women and 10 percentage points more likely than urban women to be sterilized. These differences shrink to five and six percentage points, respectively, in our fully adjusted model (Model 2), but remain statistically significant, suggesting that unobserved factors are driving the elevated rates of rural sterilization. Table S1 shows the association between women's demographic, reproductive, religious, and socioeconomic characteristics and female sterilization.

**TABLE 2:** Predicted probabilities and average marginal effects (and 95% confidence intervals) for unadjusted and adjusted binary logistic regression models of female sterilization among sexually active women aged 15–49.

	Model 1: Unadjusted				Model 2: Adjusted			
	Prob.	C.I.	A.M.E. (Sig.)	C.I.	Prob.	C.I.	A.M.E. (Sig.)	C.I.
**Residence**								
Rural	0.242	(0.200–0.283)	Ref.		0.205	(0.183–0.228)	Ref.	
Suburban	0.153	(0.132–0.174)	−0.088***	(−0.134–0.042)	0.158	(0.142–0.173)	−0.048***	(−0.074–0.021)
Urban	0.139	(0.116–0.162)	−0.102***	(0.149–0.056)	0.147	(0.129–0.165)	−0.058***	(−0.086–0.030)
	*n* = 10,081				*n* = 10,081			

*Note:* Adjusted models include control variables for current age, race/ethnicity, immigrant status, marital status, parity, unintended pregnancy, education, household income, religious affiliation, health insurance, and usual place for health care. Significance: ***p ≤ 0.001.

Abbreviations: A.M.E. = average marginal effect; C.I. = confidence interval; Prob. = predicted probability; Ref. = reference group.

### 6.3 Desire for Sterilization Reversal or More Children

Table 3 assesses whether women who have had tubal ligation want to have the procedure reversed or want to have (more) children. Among sterilized women, we find no evidence that rural women have a higher probability of desiring to reverse the procedure than suburban or urban women (Model 1). In fact, urban women (25.7%) are slightly more likely than either suburban (20.9%) or rural women (20.5%) to express a desire for sterilization reversal, but none of these differences are statistically significant. Similarly, although roughly a quarter of women who have had tubal ligation expressed the desire to have a(nother) child, there are no statistically significant differences by residence.^6^ Table 3 confirms that these findings for both desiring to have the procedure reversed and for wanting (more) children hold even after controlling for differences in the characteristics of rural, suburban, and urban women. Full results from our adjusted models are presented in Table S2.

**TABLE 3:** Predicted probabilities and average marginal effects (and 95% confidence intervals) for unadjusted and adjusted binary logistic regression models of desire for sterilization reversal or wanting a(nother) child among women with tubal ligation.

	Model 1: Unadjusted				Model 2: Adjusted			
	Prob.	C.I.	A.M.E. (sig.)	C.I.	Prob.	C.I.	A.M.E. (sig.)	C.I.
**Want sterilization reversed**								
**Residence**								
Rural	0.205	(0.135–0.275)	Ref.		0.192	(0.142–0.243)	Ref.	
Suburban	0.209	(0.165–0.253)	0.004	(−0.078–0.086)	0.226	(0.185–0.267)	0.034	(−0.032–0.099)
Urban	0.257	(0.190–0.325)	0.052	(−0.045–0.149)	0.243	(0.185–0.301)	0.051	(−0.027–0.130)
	*n* = 1260^a^				*n* = 1260^a^			
**Want a(nother) child**								
**Residence**								
Rural	0.274	(0.212–0.335)	Ref.		0.263	(0.202–0.324)	Ref.	
Suburban	0.242	(0.190–0.294)	−0.032	(−0.11–0.047)	0.255	(0.206–0.303)	−0.008	(−0.084–0.068)
Urban	0.257	(0.193–0.320)	−0.017	(−0.103–0.07)	0.247	(0.188–0.306)	−0.016	(−0.101–0.069)
	*n* = 1525				*n* = 1525			

*Note:* Adjusted models include control variables for current age, age of sterilization, race/ethnicity, immigrant status, marital status, parity, unintended pregnancy, education, household income, religious affiliation, health insurance, and usual place for health care.

Abbreviations: A.M.E. = average marginal effect; C.I. = confidence interval; Prob. = predicted probability; Ref. = reference group.

^a^ Models of desires for sterilization reversal exclude 265 women who had another sterilization procedure in addition to tubal ligation.

### 6.4 Unwanted Infecundity

**FIGURE 1:**
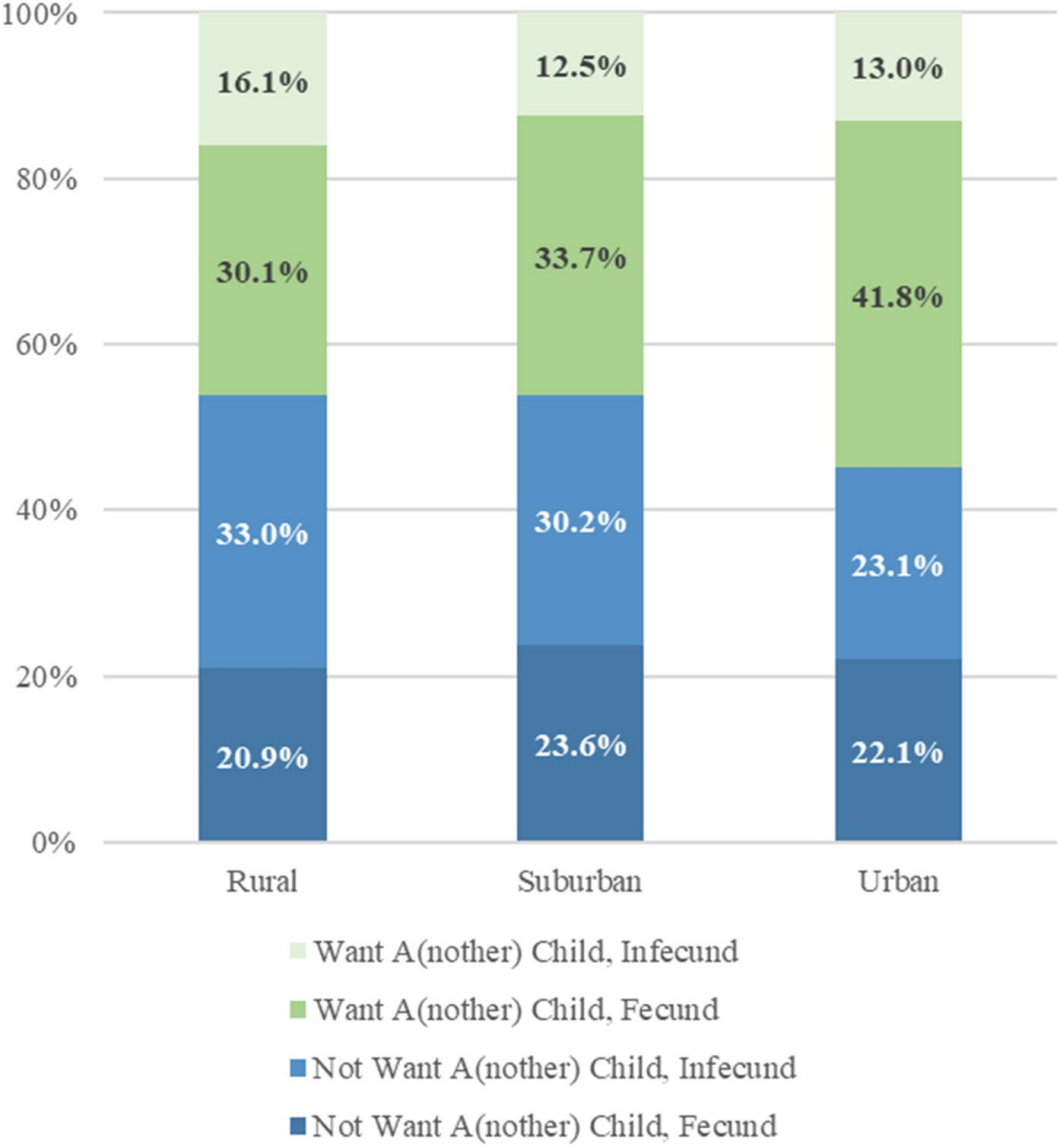
Infecundity and desire for a(nother) child. Tubal ligation excludes 12 women who had tubal ligation for non‐contraceptive purposes. Other surgical sterilization includes women who had hysterectomies, both ovaries removed, or other surgical sterilization procedures (excluding tubal ligation).

Unwanted infecundity reflects both women's fecundity and their desire for (more) children. Figure 1 shows that rural women (46.2%) are considerably less likely than urban women (54.8%, *p* = 0.005) to want a(nother) child. If risks of infecundity were equal across rural and urban areas, then one would expect more urban women to experience unwanted infecundity simply because they are more likely to want a(nother) child. Figure 1, however, shows that the proportion of *all women* who both want a(nother) child and are infecund is slightly (but not significantly) larger among rural women (16.1%) compared to suburban (12.5%, *p* = 0.059) or urban women (13.0%, *p* = 0.102). This is mainly because infecundity rates are substantially higher among rural women (49.1%) than among either suburban women (42.7%, *p* = 0.014) or urban women (36.1%, *p* < 0.000). Importantly, the likelihood of being infecund is elevated not only among rural women who do not want (more) children, but also among rural women who do want (more) children.

Table 4 further examines infecundity experienced by rural, suburban, and urban women *who want a(nother) child*. The top panel of Table 4, which presents the results for all causes of infecundity, shows that 34.8% of rural women who want a(nother) child are infecund compared to only 27.0% of suburban women and 23.8% of urban women who wish to have (more) children (Model 1). Adjusting for demographic, reproductive, religious, and socioeconomic characteristics explains about half of the rural–urban difference among women who want a(nother) child (Model 2), but this difference remains statistically significant. However, rural‐suburban differences in unwanted infecundity become statistically insignificant. The predicted probabilities of all causes of infecundity in our fully adjusted model (Model 2) show that among rural women who want a(nother) child, 29.8% are infecund compared to 25.1% of urban women. Model 1 of Table S3 shows the associations between all causes of unwanted infecundity and women's demographic, reproductive, religious, and socioeconomic characteristics.

**TABLE 4:** Predicted probabilities and average marginal effects (and 95% confidence intervals) for unadjusted and adjusted binary and multinomial logistic regression models of infecundity among women who want a(nother) child.

	Model 1: Unadjusted				Model 2: Adjusted			
	Prob.	C.I.	A.M.E. (sig.)	C.I.	Prob.	C.I.	A.M.E. (sig.)	C.I.
**Binary logit**								
***All causes of infecundity***								
**Residence**								
Rural	0.348	(0.292–0.404)	Ref.		0.298	(0.262–0.333)	Ref.	
Suburban	0.270	(0.239–0.301)	−0.078*	(−0.144–0.012)	0.276	(0.252–0.300)	−0.022	(−0.065–0.022)
Urban	0.238	(0.207–0.268)	−0.110***	(−0.174–0.046)	0.251	(0.227–0.275)	−0.046*	(−0.089–0.004)
	*n* = 5217				*n* = 5217			
**Multinomial logit**								
***Tubal ligation***								
**Residence**								
Rural	0.143	(0.110–0.176)	Ref.		0.114	(0.088–0.139)	Ref.	
Suburban	0.080	(0.060–0.101)	−0.063**	(−0.101–0.025)	0.084	(0.071–0.097)	−0.029*	(−0.058–0.001)
Urban	0.065	(0.047–0.084)	−0.078***	(−0.115–0.041)	0.070	(0.057–0.083)	−0.043**	(−0.072–0.015)
***All other causes***								
**Residence**								
Rural	0.205	(0.167–0.242)	Ref.		0.185	(0.155–0.214)	Ref.	
Suburban	0.190	(0.165–0.215)	−0.015	(−0.062–0.032)	0.192	(0.169–0.215)	0.008	(−0.031–0.046)
Urban	0.173	(0.147–0.199)	−0.032	(−0.077–0.013)	0.180	(0.157–0.204)	−0.004	(−0.041–0.032)
	*n* = 5217				*n* = 5217			

*Note:* Adjusted models include control variables for current age, race/ethnicity, immigrant status, marital status, parity, unintended pregnancy, education, household income, religious affiliation, health insurance, and usual place for health care. Significance: *p ≤ 0.05, **p ≤ 0.01, ***p ≤ 0.001.

Abbreviations: A.M.E. = average marginal effect; C.I. = confidence interval; Prob. = predicted probability; Ref. = reference group.

**FIGURE 2:**
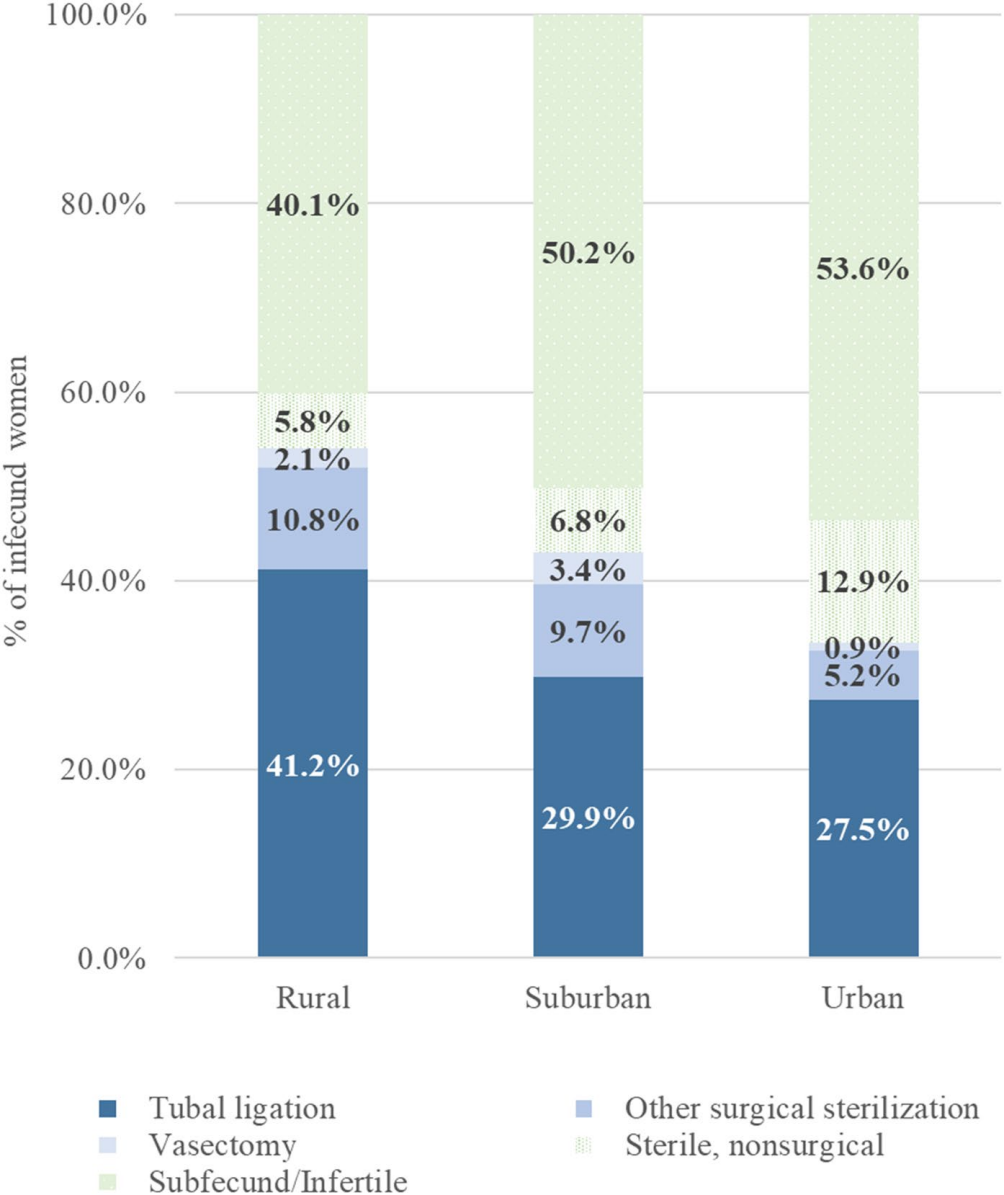
Sources of unwanted infecundity.

In addition to differences in the overall likelihood of experiencing unwanted infecundity, there may also be differences in the causes of infecundity among rural, suburban, and urban women who want to have a(nother) child. Figure 2 shows that tubal ligation accounts for a larger fraction of unwanted infecundity among rural women (41.2%) than either suburban (29.9%) or urban (27.5%) women. In contrast, urban (53.5%) and suburban (50.2%) women are substantially more likely than rural women (40.1%) to report infecundity due to infertility or subfecundity. Further, urban women are more likely than suburban or rural women to be sterile for nonsurgical reasons, but less likely to have had another surgical sterilization procedure such as a hysterectomy or bilateral ovary removal.

To determine the extent to which differences in tubal ligation are driving overall differences in unwanted infecundity, we employ a multinomial logistic regression to examine tubal ligation and all other sources of infecundity as separate outcomes (bottom panel of Table 4). In our unadjusted model (Model 1), rural women are twice as likely as urban women to be infecund because of tubal ligation (14.3% vs. 6.5%). Tubal ligation as a cause for unwanted infecundity is also 6.3 percentage points more likely among rural women than suburban women. The rural–urban and rural‐suburban difference decreases after adjusting for women's characteristics, but the gaps due to tubal ligation remain significant (Model 2). In contrast, rural, suburban, and urban women who want a(nother) child are equally likely to be infecund due to any cause other than tubal ligation. These results are consistent in both the unadjusted and adjusted models, suggesting that there are not sizable differences by residence in other causes of infecundity. Full results from our adjusted multinomial logistic regression are presented in Model 2 of Table S3.

## 7 Discussion

This study makes three main contributions. First, it updates nationally representative estimates of female sterilization across rural, suburban, and urban spaces in the United States. It documents persistent, large inequalities, particularly between rural and urban areas, and shows that these differences cannot be primarily attributed to differences in women's age, race/ethnicity, education, income, religious affiliations, type of health insurance or primary source of health care. Why then do rural women continue to rely so heavily on tubal ligation? A plausible explanation is that rural women are disproportionately more likely to opt for sterilization because other reversible methods are harder for them to access. Even if rural women have used similar types of health care (e.g., private or clinic care), the services available at these health centers may vary. Further, contraceptive methods that require prescriptions and regular visits to doctors' offices and pharmacies, such as birth control pills and emergency contraception, may be less suitable in rural areas given greater distances to these services.

Unfortunately, we are unable to use data from the NSFG to directly test for differences in the availability of contraceptive methods, nor can we investigate the specific barriers to access facing rural women. However, previous studies find that rural women are less likely than urban women to receive their contraception from health clinics that specialize in family planning or reproductive health services [38]. Rural women are also more likely to receive reproductive health care from their family physicians or nurse practitioners/physician assistants rather than from OBGYNs, who are fewer and farther between in rural areas [51]. Other studies show that rural health care providers, particularly primary care physicians, are less likely to be trained in insertion methods for LARCs, including IUDs and implants [33].

Second, it shows that rural women are not more likely than either suburban or urban women to “change their minds” and wish to have their sterilization procedure reversed or to have (more) children. Third, we demonstrate that even though rural women are not more likely to change their minds, because almost twice as many rural women as urban women undergo tubal ligation, a significantly higher fraction of rural women who want to have (more) children are infecund. Among women who wish to have (more) children, more than a third of rural women compared to less than a quarter of urban women are infecund. Further, differences in rural–urban unwanted infecundity can be largely attributed to rural women's heavier use of tubal ligation as a method of contraception. These findings provide an understudied example of how limited access to contraception methods generates a misalignment between women's fertility goals and outcomes.

### 7.1 Limitations

Despite these contributions, our paper also faces several limitations. It is challenging to define variables for a residence that capture the full range of the rural–urban continuum. One of the strengths of this paper is that it examines differences among rural, suburban, and urban women. Nonetheless, this categorical variable still masks considerable variation both across the rural–urban continuum from remote rural settings to inner city core areas and within rural areas. Despite the availability of multiple measures of rurality [52], most surveys, including the NSFG, have only one measure of rurality and restrict access to geographic data that would enable the researcher to create more nuanced measures. Similarly, cross‐sectional surveys such as the NSFG capture women's current residence. Since the history of tubal ligation is measured retrospectively, women may not have necessarily lived in a rural area at the time of sterilization.

In addition, questions which ask women whether they want to have their tubal ligation procedure reversed or want to have a(nother) child can be difficult to interpret. Although many previous studies use these questions as indicators of “regret,” [11, 12, 40, 41, 42, 43, 44] it is not clear whether women themselves would say they regret their decisions. Even women who wish to have the procedure reversed at the time of the interview may feel it was the right decision at the time. Further, although responses to questions about wanting tubal ligation reversed and wanting a(nother) child are strongly correlated among women who have had tubal ligation, they are not identical. This suggests that other factors besides fertility desires may weigh into women's assessment of desired sterilization reversal. For example, women may think the procedure would be too costly or risky. In addition, our measure of unintended infecundity captures both fertility desires and fecundity. Yet, women who are infecund may respond differently to the question about wanting a(nother) child than women who are fecund. For infecund women, this question is inherently more hypothetical, and they are likely to evaluate the potential costs and benefits of having (more) children differently than fecund women. Lastly, women's views about wanting sterilization reversal or a(nother) child are known to change over time and in light of shifting circumstances [16, 42]. Cross‐sectional studies, such as the NSFG, capture women's preferences at only one point in time.

### 7.2 Policy Implications

The American College of Obstetricians and Gynecologists (ACOG) and other health professionals have made repeated calls for expanding access to reproductive care and contraceptive methods in rural areas [1, 8, 53]. Expanded contraceptive choice, however, should not be limited to LARCs [54]. Other approaches, such as offering over‐the‐counter hormonal methods or year‐long supplies for birth control, could also significantly lessen rural women's dependence on tubal ligation by reducing the number of visits to health care providers and pharmacies [55, 56]. Efforts have begun in some rural areas to increase access to a range of contraceptive methods including LARCs, and many rural residents are enthusiastic about the prospect of telehealth to further reduce barriers to health services [57].

Nonetheless, many reproductive health researchers believe that rural women's reproductive health care will worsen significantly in response to the Supreme Court decision in Dobbs v. Jackson Women's Health Organization (Dobbs), which allowed states in the United States to ban abortion [58]. While this decision will have the most direct effect on rural women's access to abortion care, it may also reduce the number of OBGYNs practicing in rural areas and restrict the overall availability of contraceptive methods. A study published in 2017 indicates that up to one‐third of OBGYNs relocated to urban areas or areas with a lower levels of poverty within the last 10 years [59]. Following Dobbs, this percentage has likely increased, as states with abortion restrictions are already seeing a decrease in the number of practicing OBGYNs and their medical training programs are seeing a significant decline in their number of applicants [60]. At the same time, women across the country are increasingly seeking female sterilization post‐Dobbs out of fear that they will be unable to access contraception or that their contraception method will fail and they will be unable to get an abortion [36]. One study found a significant increase in the use of tubal ligation across all included states post‐Dobbs. In addition, in states that banned abortion services, there was a further monthly increase in tubal ligation after the Dobbs decision [37]. Hence, disparities in rural and urban women's dependence on tubal ligation may grow in future years.

The ability to have or not have children when desired is central to reproductive autonomy [61], which is defined as the ability of women to make reproductive decisions and to have access to reproductive health services to achieve their reproductive intention [62]. To date, most calls to improve rural women's reproductive health care and, especially, access to contraception, have argued that limited access to contraception compromises women's reproductive autonomy because it leads to unintended pregnancy and overachieved fertility. The persistence of these geographic inequalities suggests that these arguments alone have not sufficiently persuaded rural populations or rural policymakers to protect or expand access to contraception and abortion. This paper makes an important additional argument that improving the availability and range of contraceptive methods in rural areas may make it possible for rural women to have (more) wanted children.

Yet, the relationships among tubal ligation, underachieved fertility, and reproductive autonomy are complex. Although the majority of women who choose female sterilization do not express a subsequent desire to have (more) children, concerns that women will change their minds have led some healthcare professionals to refuse to provide elective sterilization to young and nulliparous women [63, 64]. Several professional associations and scholars have argued against this practice, noting that restricting access to this highly effective and safe form of birth control undermines women's reproductive autonomy and that all women should be able to choose their preferred method of birth control. This approach requires that all women have access to a range of contraceptive methods, including tubal ligation. In rural areas, limited contraceptive choice may have the unintended consequence of restricting their wanted fertility and undermining their reproductive autonomy.

## Supporting information


**Table S1.** Supporting information.

## Endnotes

^1^
For the purposes of this paper, we are using female sterilization interchangeably with tubal ligation to indicate voluntary surgical sterilization for the purposes of contraception. We have, therefore, removed 12 women who had tubal ligation for non‐contraceptive purposes.

^2^
Further analyses, which are limited to the 2017–2019 NSFG, yield substantively similar results, but our estimates are less precisely estimated. These analyses are available upon request.

^3^
The NSFG relies on respondent's self‐reported gender identities to classify respondents as male or female. Only respondents who identify as female are asked questions about pregnancy, contraception and sterilization.

^4^
This excludes four rural women, seven suburban women, and three urban women (total *n* = 14) who reported having their tubal ligation procedures reversed.

^5^
Women with 0 or 1 child are combined as less than 10% of women with tubal ligation had fewer than two children.

^6^
The correlation between women who wished to have the procedure reversed and those who wanted a(nother) child is 0.72.
