# Supplementary material for: Sterilization, Infecundity, and Reproductive Autonomy in Rural, Suburban, and Urban America: Results From a National Survey
Source: Perspect Sex Reprod Health. 2025 Mar 16;57(1):72–84. doi: 10.1111/psrh.70006 (PMC11936855; doi:10.1111/psrh.70006)
Supplement: Supplementary file 1 [file PSRH-57-72-s001.docx]

SUPPLEMENTAL TABLE 1. Average marginal effects (and 95% confidence intervals) for adjusted binary logistic regression models of female sterilization among sexually active women aged 15-49.

VARIABLES	A.M.E.(Sig.)	C.I.
Residence (ref=Rural)		
Suburban	-0.048***	(-0.074 - -0.021)
Urban	-0.058***	(-0.086 - -0.030)
Age at Interview (ref<30)		
30-34	0.064***	(0.041 - 0.087)
35-39	0.126***	(0.100 - 0.152)
40-44	0.158***	(0.127 - 0.189)
45-49	0.180***	(0.150 - 0.210)
Race/ethnicity (ref=White)		
Black	-0.003	(-0.037 - 0.030)
Hispanic	-0.002	(-0.030 - 0.026)
Other	0.013	(-0.038 - 0.065)
Immigrant	-0.050**	(-0.080 - 0.020)
Union Status (ref=Married)		
Cohabiting	0.010	(-0.023 - 0.043)
Formerly married	-0.002	(-0.031 - 0.027)
Never married	-0.042*	(-0.078 - -0.006)
Parity (ref= 0-1 Children)		
2 Children	0.164***	(0.141 - 0.187)
3 Children	0.249***	(0.207 - 0.292)
>=4 Children	0.262***	(0.215 - 0.310)
Unwanted pregnancy	0.042***	(0.020 - 0.065)
Education (ref<High school)		
High school degree	-0.006	(-0.037 - 0.024)
Some college	-0.014	(-0.048 - 0.020)
B.A. or higher	-0.073***	(-0.104 - -0.041)
Household income (ref=<$25,000)		
$25,000-<$50,000	-0.027	(-0.057 - 0.004)
$50,000-<$$75,000	-0.030	(-0.067 - 0.007)
>=$75,000	-0.071***	(-0.112 - -0.031)
Religious Affiliation (ref=No religion)		
Christian, non-fundamentalist	-0.004	(-0.030 - 0.023)
Christian, fundamentalist	0.032*	(0.003 - 0.061)
Other religion	-0.009	(-0.062 - 0.044)
Insurance (ref=Not covered)		
Public	0.017	(-0.010 - 0.044)
Private	0.018	(-0.014 - 0.049)
Usual health care (ref=None)		
HMO/private	0.012	(-0.022 - 0.045)
Clinic	0.016	(-0.020 - 0.053)
Emergency care	0.025	(-0.019 - 0.069)
	n=10,081	

Significance: *p<=0.05, **p<=0.01, ***p<=0.001

Abbreviations: ref=reference group; A.M.E.=average marginal effect; C.I.=confidence interval.

SUPPLEMENTAL TABLE 2. Average marginal effects (and 95% confidence intervals) for adjusted binary logistic regression models of desire for reversal and wanting a(nother) child among women with tubal ligation.

	Model 1: Desire Sterilization Reversal		Model 2: Want A(nother) Child	
VARIABLES	A.M.E.(Sig.)	C.I.	A.M.E.(Sig.)	C.I.
Residence (ref=Rural)				
Suburban	0.034	(-0.032 - 0.099)	-0.008	(-0.084 - 0.068)
Urban	0.051	(-0.027 - 0.130)	-0.016	(-0.101 - 0.069)
Age at Interview (ref<30)				
30-34	-0.136	(-0.277 - 0.005)	-0.210**	(-0.338 - -0.083)
35-39	-0.214**	(-0.351 - -0.077)	-0.246***	(-0.374 - -0.118)
40-44	-0.254***	(-0.389 - -0.119)	-0.326***	(-0.454 - -0.199)
45-49	-0.378***	(-0.505 - -0.251)	-0.381***	(-0.509 - -0.254)
Age at Sterilization (ref<25)				
25-29	-0.006	(-0.083 - 0.072)	-0.004	(-0.082 - 0.073)
30-34	-0.023	(-0.110 - 0.063)	-0.011	(-0.096 - 0.073)
>=35	-0.055	(-0.171 - 0.060)	-0.040	(-0.138 - 0.059)
Race/ethnicity (ref=White)				
Black	0.041	(-0.043 - 0.125)	0.006	(-0.077 - 0.088)
Hispanic	0.090	(-0.007 - 0.186)	0.117*	(0.028 - 0.207)
Other	0.237**	(0.063 - 0.412)	0.120	(-0.004 - 0.243)
Immigrant	-0.129***	(-0.205 - -0.054)	-0.119**	(-0.202 - -0.036)
Union Status (ref=Married)				
Cohabiting	0.044	(-0.044 - 0.132)	0.105*	(0.006 - 0.205)
Formerly married	0.060	(-0.032 - 0.152)	0.019	(-0.062 - 0.099)
Never married	-0.036	(-0.119 - 0.047)	0.060	(-0.058 - 0.179)
Parity (ref= 0-1 Children)				
2 Children	-0.046	(-0.198 - 0.106)	-0.103	(-0.227 - 0.021)
3 Children	-0.027	(-0.187 - 0.133)	-0.040	(-0.190 - 0.110)
>=4 Children	0.042	(-0.119 - 0.203)	-0.058	(-0.202 - 0.087)
Unwanted pregnancy	-0.042	(-0.108 - 0.024)	-0.069	(-0.140 - 0.003)
Education (ref<High school)				
High school degree	0.046	(-0.021 - 0.112)	0.011	(-0.059 - 0.081)
Some college	0.039	(-0.039 - 0.117)	0.009	(-0.073 - 0.091)
B.A. or higher	-0.036	(-0.144 - 0.073)	-0.019	(-0.114 - 0.077)
Household income (ref=<$25,000)				
$25,000-<$50,000	-0.007	(-0.095 - 0.080)	-0.047	(-0.126 - 0.032)
$50,000-<$$75,000	-0.009	(-0.120 - 0.102)	0.018	(-0.089 - 0.124)
>=$75,000	-0.090	(-0.195 - 0.014)	-0.044	(-0.153 - 0.064)
Religious Affiliation (ref=No religion)				
Christian, non-fundamentalist	0.079*	(0.002 - 0.156)	0.111**	(0.028 - 0.194)
Christian, fundamentalist	0.089**	(0.026 - 0.152)	0.116***	(0.054 - 0.178)
Other religion	0.046	(-0.098 - 0.190)	-0.105*	(-0.193 - -0.017)
Insurance (ref=Not covered)				
Public	-0.041	(-0.101 - 0.018)	-0.006	(-0.079 - 0.068)
Private	0.067	(-0.032 - 0.165)	0.030	(-0.069 - 0.129)
Usual health care (ref=None)				
HMO/private	-0.003	(-0.071 - 0.064)	0.027	(-0.059 - 0.114)
Clinic	0.057	(-0.033 - 0.148)	0.071	(-0.030 - 0.172)
Emergency care	0.095	(-0.042 - 0.232)	0.053	(-0.093 - 0.199)
	n=1,260[a]		n=1,525	

Significance: *p<=0.05, **p<=0.01, ***p<=0.001

Abbreviations: ref=reference group; A.M.E.=average marginal effect; C.I.=confidence interval.

a: Models of desires for sterilization reversal exclude 265 women who had another sterilization procedure in addtion to tubal ligation.

SUPPLEMENTAL TABLE 3. Average marginal effects (and 95% confidence intervals) for adjusted binary and multinomial logistic regression models of infecundity among women who want a(nother) child.

	Model 1: Binary Logit		Model 2: Multinomial Logit			
	All Infecundity		*Infecundity due to Tubal Ligation*		*Infecundity All Other Causes*	
VARIABLES	A.M.E.(Sig.)	C.I.	A.M.E.(Sig.)	C.I.	A.M.E.(Sig.)	C.I.
Residence (ref=Rural)						
Suburban	-0.022	(-0.065 - 0.022)	-0.029*	(-0.058 - -0.001)	0.008	(-0.031 - 0.046)
Urban	-0.046*	(-0.089 - -0.004)	-0.043**	(-0.072 - -0.015)	-0.004	(-0.041 - 0.032)
Age at Interview (ref<30)						
30-34	0.086***	(0.043 - 0.129)	0.026*	(0.004 - 0.049)	0.060**	(0.021 - 0.099)
35-39	0.241***	(0.182 - 0.300)	0.071***	(0.045 - 0.096)	0.166***	(0.107 - 0.224)
40-44	0.418***	(0.343 - 0.492)	0.113***	(0.072 - 0.154)	0.299***	(0.227 - 0.371)
45-49	0.567***	(0.480 - 0.654)	0.136***	(0.083 - 0.189)	0.423***	(0.329 - 0.517)
Race/ethnicity (ref=White)						
Black	-0.051*	(-0.097 - -0.005)	-0.001	(-0.024 - 0.022)	-0.050*	(-0.091 - -0.010)
Hispanic	-0.031	(-0.075 - 0.012)	0.016	(-0.006 - 0.037)	-0.046*	(-0.082 - -0.010)
Other	0.003	(-0.081 - 0.087)	0.002	(-0.047 - 0.052)	-0.004	(-0.086 - 0.079)
Immigrant	-0.055*	(-0.098 - -0.013)	-0.046***	(-0.066 - -0.025)	-0.012	(-0.051 - 0.027)
Union Status (ref=Married)						
Cohabiting	-0.056*	(-0.107 - -0.005)	-0.021	(-0.045 - 0.002)	-0.036	(-0.082 - 0.010)
Formerly married	-0.055	(-0.119 - 0.010)	0.002	(-0.024 - 0.029)	-0.069*	(-0.127 - -0.011)
Never married	-0.153***	(-0.195 - -0.111)	-0.026	(-0.053 - 0.001)	-0.128***	(-0.168 - -0.089)
Parity (ref= 0-1 Children)						
2 Children	0.004	(-0.031 - 0.040)	0.095***	(0.069 - 0.121)	-0.091***	(-0.129 - -0.053)
3 Children	0.119***	(0.058 - 0.181)	0.215***	(0.160 - 0.270)	-0.115***	(-0.158 - -0.071)
>=4 Children	0.158**	(0.057 - 0.259)	0.228***	(0.155 - 0.302)	-0.097*	(-0.171 - -0.023)
Unwanted pregnancy	0.123***	(0.082 - 0.164)	0.033**	(0.012 - 0.055)	0.083***	(0.046 - 0.121)
Education (ref<High school)						
High school degree	-0.033	(-0.086 - 0.020)	-0.007	(-0.027 - 0.014)	-0.024	(-0.081 - 0.032)
Some college	-0.020	(-0.076 - 0.035)	-0.012	(-0.038 - 0.014)	-0.007	(-0.065 - 0.051)
B.A. or higher	-0.088**	(-0.147 - -0.028)	-0.037**	(-0.062 - -0.012)	-0.052	(-0.113 - 0.009)
Household income (ref=<$25,000)						
$25,000-<$50,000	-0.030	(-0.076 - 0.015)	-0.025	(-0.053 - 0.004)	-0.003	(-0.053 - 0.047)
$50,000-<$$75,000	-0.050*	(-0.096 - -0.004)	-0.004	(-0.038 - 0.031)	-0.045	(-0.093 - 0.003)
>=$75,000	-0.070*	(-0.128 - -0.012)	-0.032*	(-0.063 - -0.001)	-0.039	(-0.092 - 0.015)
Religious Affiliation (ref=No religion)						
Christian, non-fundamentalist	0.003	(-0.036 - 0.041)	0.005	(-0.022 - 0.033)	-0.000	(-0.037 - 0.036)
Christian, fundamentalist	0.063**	(0.020 - 0.107)	0.027*	(0.006 - 0.049)	0.037	(-0.002 - 0.077)
Other religion	0.007	(-0.100 - 0.113)	-0.038	(-0.080 - 0.005)	0.033	(-0.070 - 0.136)
Insurance (ref=Not covered)						
Public	0.022	(-0.023 - 0.067)	0.001	(-0.021 - 0.023)	0.023	(-0.023 - 0.068)
Private	0.023	(-0.038 - 0.084)	0.003	(-0.027 - 0.034)	0.021	(-0.044 - 0.087)
Usual health care (ref=None)						
HMO/private	0.061*	(0.014 - 0.108)	0.011	(-0.020 - 0.042)	0.048*	(0.000 - 0.096)
Clinic	0.061*	(0.007 - 0.116)	0.028	(-0.008 - 0.065)	0.033	(-0.028 - 0.095)
Emergency care	0.151***	(0.078 - 0.224)	0.035	(-0.007 - 0.077)	0.112**	(0.036 - 0.188)
	n=5,217		n=5,217			

Significance: *p<=0.05, **p<=0.01, ***p<=0.001

Abbreviations: ref=reference group; A.M.E.=average marginal effect; C.I.=confidence interval.
